# Human rights engagement, stigma and attitudes towards mental health among Colombian social work and medical students

**DOI:** 10.1007/s10459-024-10377-5

**Published:** 2024-11-06

**Authors:** Felipe Agudelo-Hernández, Helena Vélez-Botero, Marcela Guapacha-Montoya

**Affiliations:** 1 Universidad de Manizales, Manizales, Caldas Colombia; 2 Universidad Nacional, Bogotá, Colombia; 3 Universidad de Caldas, Manizales, Caldas Colombia

**Keywords:** Mental health, Human rights, Competence, professional, Attitude of health personnel

## Abstract

Human rights training in mental health professions is essential to reduce stigma and facilitate recovery processes. The aim of this study was to understand the relationship between engagement to human rights and stigma towards people with mental disorders, as well as attitudes towards mental illness in medical and social work students of a Latin American context. An analytical, cross-sectional study was carried out with a sample of 243 students in the last two years of study. Community Attitudes Towards the Mentally Ill (CAMI), Mental Illness Clinicians’ Attitude Scale (MICA), Human Rights Exposure in Social Work (HRXSW) and Human Rights Engagement in Social Work (HRESW) were used as instruments. To determine how the explanatory variables are related to the engagement to human rights (dependent variable) a binary logistic regression model was used. Correlations were found between the scales and their components. Economic condition, intersectional approach, authoritarianism, benevolence and mental health ideology explained 55.11% of the variance for commitment to the engagement of human rights. Some factors related to the stigma of mental disorders and the focus on human rights in future mental health professionals are highlighted that should be more visible in the training practices of these professions.

## Introduction

The primary goal of this education is to equip individuals with the knowledge, skills, and values necessary to recognize, claim, and defend their rights. The essential and typically evident contribution lies in the increase of knowledge through the provision of information and awareness (Dziva et al., 2014; Gostin et al., 2020; Hagenaars, 2016; Mann et al., 2016). This awareness of human rights issues allows individuals to understand both their rights and the rights of others (Gill, 2018).

Additionally, human rights education in health professions helps professionals understand the social determinants of health and how they intersect with human rights (Gostin et al., 2020; Newham et al., 2021). Some studies have indicated that some sociodemographic factors have been associated with the perception of people with mental disorders (Sayeed et al., 2020). These include socioeconomic status, gender, or having a chronic illness (Lewis, 2023; Sayeed et al., 2020).

The knowledge of principles such as equality, non-discrimination, dignity, and respect provide individuals with the necessary tools to recognize violations and advocate for the protection of human rights (Dziva et al., 2014). This, in turn, promotes critical thinking skills by encouraging individuals to analyze human rights issues from various perspectives and challenge social norms.

Human rights training promoting a culture of respect and dignity (Dziva et al., 2014; Newham et al., 2021). Additionally, the fosters empathy and understanding by exposing individuals to the experiences of those who have suffered discrimination or violence and helps them better understand the impact of human rights violations on individuals and communities (Dziva et al., 2014; Gostin et al., 2020; Tibbitts, 2017).

In the realm of healthcare education, understanding the intricate dynamics between human rights principles and attitudes towards individuals with mental illness is paramount (Gostin et al., 2020). Human rights education plays a significant role in reducing the stigma associated with mental health conditions by providing accurate information about mental health, debunking myths, and replacing misconceptions with facts (MacSorley et al., 2023). This shift in perspective can lead to reduced stigma and more supportive attitudes toward individuals with mental health conditions (Moudatsou et al., 2020). By promoting awareness, challenging stereotypes, and fostering inclusive attitudes, human rights education contributes to creating a more compassionate and understanding society (Moudatsou et al., 2020).

Regarding the various professions involved in healthcare have more effectively incorporated the significance of human rights into their practices and curricula (Ruth & Marshall, 2017). This enables them to comprehend, identify, and address the structural and contextual factors rooted in the environments where populations reside (Ross et al., 2020). Social work training employs competency-based approaches structured around engagement, assessment, intervention, and evaluation in all individual and group systems, which encompass human rights both in knowledge and in the commitment to promote and safeguard them (Cubillos et al., 2019). Therefore, healthcare professionals are guided by a commitment to prevention, health promotion, and advocacy for human rights. They employ integrated, transdisciplinary approaches to promote health equity and mitigate human health issues (Cubillos et al., 2019; Ross et al., 2020). In the field of health of sciences, for instance, education has empowered healthcare personnel to use the language of rights, such as advocating for patients’ rights to informed consent and privacy, to effect health-related changes (Newham et al., 2021; Riffel & Chen, 2020).

At the Latin American level, the impact of human rights education on health professions is not precisely defined. However, it is recognized that more concepts about inequities, understanding of contexts, and social determinants are increasingly being included in public health subjects (Agudelo-Hernández et al., 2024; Clay et al., 2020; Suárez Conejero et al., 2023). The objective of human rights education is to generate a culture of respect for others, considering all visualized differences (Newham et al., 2021), this is in line with the foundations of medicine and social work.

People with mental health problems often do not have access to quality services, are subjected to coercive practices, inhumane treatment, and, in some cases, abuse, even in healthcare settings, where they should be protected (Gill et al., 2024). In relation to the above, psychiatric practice must currently understand the context and the individual meaning of each person who is part of society (Tibbitts, 2017). The focus on human rights as a principle and quality tool in health can contribute to a more humanized medical practice, a holistic view of the person including individual contexts and cultures, as well as ways of coping with life situations (Clay et al., 2020).

Systematic reviews on strategies to reduce stigma point to few studies in Latin America (Clay et al., 2020; Agudelo-Hernández & Urrego-García, 2024). In the field of health education, it is essential to understand the intricate dynamics between human rights and stigma towards people with mental illness. Despite having curricula with elements to reduce stigma and having an appropriate attitude towards mental health knowledge, a stigmatizing attitude persists in health professions (Riffel & Chen, 2020) that hinders recovery (Gill et al., 2024; Patel et al., 2018). For this reason, the objective of the present study was to understand the relationship between engagement to human rights and stigma towards people with mental disorders, as well as attitudes towards mental illness in medical and social work students of a Colombian context. The above, in students in the last two years of study. It is hypothesized that engagement with human rights in medical and social work students is influenced by the students’ perception of people with mental disorders, by their attitude towards mental health training, and by the students’ notion of these rights, as well as by some sociodemographic variables.

The purpose is to contribute to the understanding of the factors that influence the engagement to the practice of human rights, beyond theoretical elements, that can modify stigmatizing practices in mental health professionals. The above, to improve the recovery processes of people with mental disorders within a framework of human rights that reaffirm the foundations of their respective professions.

## Methods

### Type of study and participants

A cross-sectional study was conducted using a simple random sampling method over the last three years of the social work and medicine programs of two universities in Colombia. The choice of these universities arose from a criterion of opportunity, since both programs offered their collaboration with the study. In this way, 243 students were included.

The self-report of the students regarding the presence of health problems in the students is also shown. Aspects related to socioeconomic status, health backgrounds, and the importance assigned to mental health by participants were investigated, on a scale from one to 10 (Table 1).

**Table 1:** Sociodemographic data

Gender	%
Female	70.4
Male	29.5
Academic Program	
Medicine	65.8
Social Work	34.2
Medical History	
None	70
Cardiovascular	1.2
Respiratory	4.1
Neurological	1.6
Psychiatric	13.6
Endocrine	2.1
Osteomuscular	0.4
Sensory	0.4
Consumption of psychoactive substances	1.2
Gastrointestinal	3.7
Autoinmune	1.2
Cancer	0.4
Socioeconomic condition	
Very low	0.4
Low	3.3
Medium	53.1
Medium high	14.8
High	12.8
Very high	15.6

## Measures

### Human rights exposure in social work (HRXSW) / human rights engagement in social work (HRESW)

The HRXSW was initially developed to measure the knowledge and exposure of social work students to human rights, while the HRESW addresses whether students understand social work practice within a human rights framework (McPherson & Abell, 2012). These scales have been used in other countries (Chen & Tang, 2019; Kwon et al., 2017; Tuggle et al., 2023). The HRXSW scale consists of 11 items and was developed to measure familiarity with human rights principles and concerns. The HRESW scale, which contains 25 items, determines commitment to human rights. The latter, in its initial version, contains three domains: support for human rights principles, perceived relevance of human rights to professional practice, and application of these concepts in practice (McPherson & Abell, 2012). The instruments were initially validated with 287 social work students from the United States (McPherson & Abell, 2012), and their validation into Spanish was carried out with 498 students, maintaining adequate psychometric properties (Cubillos et al., 2019).

In its translation and validation to a Spanish-speaking context, 16 items were proposed for HRXSW and 25 items for HRESW, with Likert-type response options showing the degree of agreement with the statement (1 = extremely disagree and 7 = extremely agree) (Cubillos et al., 2019). Cubillos et al. (2019) obtained in the Spanish validation a Cronbach’s alpha of 0.803 for the NDHTS scale and an alpha of 0.856 for CDHTS.

## Community attitudes towards the mentally ill (CAMI)

The CAMI scale is designed to measure the degree of acceptance or rejection towards people with mental illnesses, as well as to explain the community’s reaction to environments where mental health services are provided (Link et al., 2004). It has been validated in different contexts, specifically in Latin America (Abelha et al., 2015) and Colombia (Pinto, 2020).

It evaluates four factors: authoritarianism, benevolence, social restriction, and mental health ideology. Each subdomain has five statements formulated positively and five negatively. It is answered with Likert options ranging from completely agree to completely disagree, from one to five. In the initial scale, internal consistency reports by subscale showed a Cronbach’s alpha of 0.88 in health ideology, 0.80 in social restriction, 0.76 in benevolence, and 0.68 in authoritarianism (Link et al., 2004). In the Colombian validation, these values ranged from 0.59 to 0.80 (Pinto, 2020).

## Mental Illness clinicians’ attitude scale (MICA)

The MICA Scale evaluates attitudes towards mental illness among students or staff from any health discipline and is a 16-item scale (Gabbidon et al., 2013). Each item requires a response on a 6-point Likert scale (“strongly agree, agree, somewhat agree, somewhat disagree, disagree, strongly disagree”). A single overall score is calculated by adding each individual item where a high overall score indicates more negative stigmatizing attitudes, with a possible range of 16–96. In its initial version, it reported good internal consistency (α = 0.79). The standardized response mean for the scale was 0.4 (95% CI 0.02–0.8) after a mental illness-related stigma intervention (Kassam et al., 2010). Adaptations to Latin American contexts have preserved psychometric properties (Vistorte et al., 2023).

## Procedure

At all times, necessary measures were taken to respect people’s privacy by coding the information when systematized. The original forms were kept in secure and confidential conditions only by the lead researchers. Clear and truthful information about the purpose, objective of the study, and the meaning of their participation was offered to each subject, which was corroborated by signing an informed consent form. Although the research objectives were clearly stated, there was the possibility of finding alterations that imply a high risk of affecting the participant’s integrity or their environment. Therefore, a care pathway was activated by contacting University Health services to initiate the same, and a group meeting for psychoeducation and route signalling was also conducted.

This work complies with research standards in human beings as provided for in Resolution No. 008430 of 1993 of the Ministry of Health and in the Helsinki Declaration of 2000. It is a minimum risk investigation, according to the Bioethics Committee [masked].

### Analysis

Statistical analysis was performed using SPSS 26. Antes de las pruebas estadísticas se examinó la normalidad con la prueba de Shapiro-Wilk. When comparing two means, Mann-Whitney U test was used. Kruskal-Wallis tests were conducted to evaluate potential differences in study variables concerning different grouping variables, such as personal or family history of illness or disability, and opinions regarding mental health, the role of other disciplines, and the care of individuals with mental health issues. Due to the lack of cut-off points on the scale that determines engagement, the 50th Percentile of the HRESW Scale was used to convert engagement with human rights into a dichotomous variable and use it as a dependent variable. This percentile was close to the averages of other studies for this scale that determined adequate human rights engagement based on these averages (Chen & Tang, 2019; Cubillos et al., 2019; Tuggle et al., 2023).

Subsequently, a binary logistic regression was applied that established the relationship between the dependent variable (engagement with human rights) and the other variables, through an explanatory model. The initial model showed the influence of some sociodemographic variables, exposure to human rights, stigma, and students’ attitudes toward mental health on engagement with human rights. Variables with the weakest associations were eliminated at each step using the backward method, until those with significance level *p* <.05 remained in the final model.

## Results

Students rated the importance they give to mental health in their lives on a scale from 1 to 10. Based on this evaluation, significant differences were observed in human rights notions and engagement to human rights, indicating that those who prioritize their mental health have higher levels of knowledge and commitment. It was observed that human rights notions, engagement to them. The favourable attitude towards psychiatry education is higher among those in agreement with the strategic alliance between social sciences, communities, and mental health professionals. Whereas authoritarianism is higher among those who disagree with this strategic alliance. The contrast based on belief in equal attention between mental and physical health shows that greater favourability towards this belief corresponds to higher levels of human rights knowledge, engagement to them and willingness to teach and learn psychiatry (Table 2).

**Table 2:** Rank Test based on beliefs

Grouping variables		HRXSW		HRESW		CAMI		Authoritar		Benevolenc		Social restriction		Mental health ideology	
		N	Averange rank	N	Averange rank	N	Averange rank	N	Averange rank	N	Averange rank	N	Averange rank	N	Averange rank
How important do you consider promoting mental health in your daily life?	1 (None)	3	125.33	3	179.50	3	146.33	3	95.83	3	97.17	3	109.33	3	21.17
	2	2	96.00	2	100.00	2	192.25	2	136.00	2	109.75	2	83.25	2	98.75
	3	2	100.75	2	30.00	2	75.25	2	73.00	2	90.50	2	163.75	2	73.00
	4	1	70.50	1	96.50	1	158.50	1	141.50	1	159.00	1	191.50	1	31.50
	5	13	85.23	13	130.58	13	112.23	13	119.31	13	113.46	13	103.31	13	135.73
	6	14	124.75	14	97.57	14	113.64	14	135.25	14	110.32	14	118.89	14	122.43
	7	31	116.03	31	96.87	31	120.32	31	118.06	31	126.03	31	115.89	31	131.90
	8	34	111.87	34	107.94	34	144.25	34	122.50	34	126.71	34	126.12	34	128.72
	9	20	86.20	20	96.38	20	118.53	20	139.95	20	108.58	20	123.98	20	110.15
	10 (A lot)	123	136.80	123	138.92	123	117.55	123	119.76	123	125.11	123	124.11	123	122.44
	*Kruskal-Wallis H*		*16.159*		*22.764*		*7.941*		*3.611*		*2.955*		*3.857*		*11.129*
	*df*		*9*		*9*		*9*		*9*		*9*		*9*		*9*
	*Sig.*		*0.064*		*0.007*		*0.540*		*0.935*		*0.966*		*0.921*		*0.267*
Social sciences and communities can be strategic allies for developing better mental health processes	CompletDisagree	4	44.13	4	51.63	4	7.75	4	110.88	4	83.38	4	128.38	4	147.50
	Disagree	3	61.33	3	17.50	3	10.17	3	128.83	3	151.83	3	103.17	3	100.83
	Partially Disagree	9	77.61	9	61.83	9	104.61	9	122.50	9	93.83	9	120.72	9	114.44
	Partially Agree	28	105.82	28	76.50	28	134.55	28	140.61	28	126.70	28	132.14	28	119.11
	Agree	58	118.46	58	89.55	58	127.97	58	142.56	58	131.11	58	137.06	58	126.43
	CompletAgree	141	133.00	141	152.44	141	123.78	141	109.99	141	119.58	141	114.09	141	120.96
	*Kruskal-Wallis H*		*15.825*		*67.813*		*20.149*		*11.243*		*4.518*		*5.344*		*1.223*
	*df*		*5*		*5*		*5*		*5*		*5*		*5*		*5*
	*Sig.*		*0.007*		*< 0.001*		*< 0.001*		*0.047*		*0.477*		*0.375*		*0.943*
People with mental health issues should receive the same level of care as those with physical problems	CompletDisagree	5	87.70	5	72.10	5	30.90	5	117.10	5	102.20	5	105.00	5	129.40
	Disagree	5	36.30	5	92.60	5	108.50	5	139.10	5	133.90	5	126.50	5	149.30
	Partially Disagree	10	80.55	10	73.05	10	106.30	10	112.50	10	132.85	10	147.40	10	102.70
	Partially Agree	18	96.97	18	82.81	18	149.64	18	152.53	18	139.72	18	146.47	18	140.00
	Agree	49	123.38	49	78.43	49	119.31	49	141.00	49	127.72	49	137.12	49	121.95
	CompletAgree	156	130.96	156	145.89	156	124.02	156	112.73	156	117.71	156	113.20	156	120.06
	*Kruskal-Wallis H*		*16.941*		*50.714*		*12.086*		*10.257*		*2.864*		*8.615*		*2.891*
	*df*		*5*		*5*		*5*		*5*		*5*		*5*		*5*
	*Sig.*		*0.005*		*< 0.001*		*0.034*		*0.068*		*0.721*		*0.125*		*0.717*

** The correlation is significant at the 0.01 level (bilateral). C = Correlation coefficient. Sig = Bilateral significance

Regarding knowledge of human rights (HRXSW) and engagement to their defence, significant differences were found indicating a higher conceptual mastery and commitment among women and social work students. For the attitude variable (“MICA”), medical students showed a better disposition towards the study and teaching of psychiatry. Similar findings were observed regarding “Authoritarianism,” indicating a greater inclination among medical students towards this approach in healthcare. Differences were also observed in the “Ideology of Mental Health” variable, with men showing higher scores, indicating a favourable disposition towards mental health (Table 3).

**Table 3:** Rank test based on sociodemographic variables

Study variables	Grouping variables	*N*	Average rank	Sum of rank	U Mann-whitney	W Wilcoxon	Z	Significance (two-tailed)
HRXSW	Female	171	128.95	22051.00	4967.000	7595.000	-2.377	0.017
	Male	72	105.49	7595.00				
HRESW	Female	171	127.89	21870.00	5148.000	7776.000	-2.015	0.044
	Male	72	108.00	7776.00				
CAMI	Female	171	116.48	19917.50	5211.500	19917.500	-1.889	0.059
	Male	72	135.12	9728.50				
Authoritarianism	Female	171	115.97	19830.50	5124.500	19830.500	-2.068	0.039
	Male	72	136.33	9815.50				
Benevolence	Female	171	115.42	19737.00	5031.000	19737.000	-2.263	0.024
	Male	72	137.63	9909.00				
Social Restriction	Female	171	117.93	20165.50	5459.500	20165.500	-1.400	0.161
	Male	72	131.67	9480.50				
Mental Health Ideology	Female	171	115.44	19741.00	5035.000	19741.000	-2.252	0.024
	Male	72	137.57	9905.00				
HRXSW	Medicine	160	111.77	17883.50	5003.500	17883.500	-3.150	0.002
	Social Work	83	141.72	11762.50				
HRESW	Medicine	160	120.66	19305.00	6425.000	19305.000	-0.414	0.679
	Social Work	83	124.59	10341.00				
CAMI	Medicine	160	130.29	20847.00	5313.000	8799.000	-2.556	0.011
	Social Work	83	106.01	8799.00				
Authoritarianism	Medicine	160	129.66	20745.50	5414.500	8900.500	-2.366	0.018
	Social Work	83	107.23	8900.50				
Benevolence	Medicine	160	127.84	20454.50	5705.500	9191.500	-1.810	0.070
	Social Work	83	110.74	9191.50				
Social Restriction	Medicine	160	125.34	20055.00	6105.000	9591.000	-1.036	0.300
	Social Work	83	115.55	9591.00				
Mental Health Ideology	Medicine	160	122.85	19655.50	6504.500	9990.500	− 0.262	0.793
	Social Work	83	120.37	9990.50				
MICA	Medicine	160	56.96	18610.50	6504.500	8987.500	− 0.262	0.018
	Social Work	83	53.89	8915.80	5484.89			

** The correlation is significant at the 0.01 level (bilateral). C = Correlation coefficient. Sig = Bilateral significance

No significant differences were observed based on students’ current year of training, and few were related to personal health issues. Results suggest differences only in the ideology of mental health regarding the presence of different types of health difficulties or disabilities. Similarly, based on the presence or absence of health difficulties or disabilities in the family, only a significant difference was observed in the perception of social restriction. However, concerning other variables related to students’ beliefs and attitudes, significant differences of interest were observed.

Table 4 shows the correlations between the variables. Statistically significant and negative correlations are found between the Socioeconomic condition and notions of human rights. This difference is positive in the MICA scales and in the domains of Authoritarianism, Benevolence and Ideology of mental health, of the CAMI Scale. Likewise, correlations are found between the MICA Scale and the intersectional approach domain of the Engagement to Human Rights Scale, and with all the variables of the CAMI Scale.

**Table 4:** Correlations between the variables

	MH	Academy	General	Total HRXSW	Defense	Health	Total HRESW	Total MICA	Author	Benev	Restric	Ideol
Economic	0.068	− 0.089	− 0.167**	− 0.153*	− 0.107	0.01	− 0.025	0.600**	0.215**	0.199**	0.119	0.140*
Sig	0.293	0.167	0.009	0.017	0.097	0.881	0.697	<0.001	0.001	0.002	0.064	0.029
MH	1	0.094	0.185**	0.172**	0.113	0.031	0.091	0.035	− 0.084	− 0.082	− 0.132*	− 0.136*
Sig		0.146	0.004	0.007	0.078	0.627	0.155	0.586	0.19	0.204	0.04	0.034
Academy		1	0.712**	0.905**	0.386**	0.268**	0.345**	− 0.052	− 0.055	− 0.044	− 0.064	0.109
Sig			<0.001	<0.001	<0.001	<0.001	<0.001	0.418	0.393	0.499	0.323	0.09
General			1	0.935**	0.313**	0.249**	0.320**	− 0.048	− 0.008	0.044	0.04	0.171**
Sig				<0.001	<0.001	<0.001	<0.001	0.458	0.899	0.497	0.535	0.007
Total HRXSW				1	0.386**	0.278**	0.365**	− 0.053	− 0.042	0.003	− 0.019	0.147*
Sig					<0.001	<0.001	<0.001	0.408	0.51	0.961	0.763	0.022
Defense					1	0.632**	0.866**	− 0.004	− 0.206**	0.043	− 0.210**	0.008
Sig						<0.001	<0.001	0.946	0.001	0.501	0.001	0.899
Health						1	0.875**	0.081	− 0.067	0.127*	− 0.089	0.064
Sig							<0.001	0.21	0.296	0.048	0.168	0.32
Total HRESW							1	0.109	− 0.097	0.117	− 0.106	0.093
Sig								0.089	0.133	0.068	0.1	0.15
Total MICA								1	0.309**	0.320**	0.253**	0.259**
Sig									<0.001	<0.001	<0.001	<0.001
Authoritarianism									1	0.412**	0.403**	0.490**
Sig										<0.001	<0.001	<0.001
Benevolence										1	0.373**	0.335**
Sig											<0.001	<0.001
Restriction											1	0.375**
Sig												<0.001

MH = Medical History. ** The correlation is significant at the 0.01 level (bilateral). C = Correlation coefficient. Sig = Bilateral significance

Table 5 shows the binary logistic regression model using the intro method to determine the role that human rights concepts have in training, some sociodemographic variables and the community perception of mental disorders, associated with the variance of the attitude towards mental disorders. Those previously described were used as independent variables, which showed correlation with the MICA Scale. The model obtained presented a good fit (Hosmer and Lemeshow chi = 19.280; df = 6; *p* =.016). The estimated model shows that the independent variables presented explain 55.11% of the variance. The log-likelihood-2 was 99.99. The B coefficient was positive for stratum, intersectional approach, authoritarianism, benevolence and mental health ideology.

**Table 5:** Binary logistic regression analysis

Variable	B	SE	Wald	Df	Sig.	OR
Economy	2.562	0.347	54.408	1	< 0.001	12.960
Intersectional approach	0.060	0.069	0.758	1	0.384	1.062
Authoritarianism	0.133	0.092	2.074	1	0.150	1.142
Benevolence	0.086	0.103	0.691	1	0.406	1.089
Social Restriction	− 0.104	0.086	1.444	1	0.229	0.902
Ideology	0.004	0.097	0.002	1	0.968	1.004
Constant	-16.419	2.791	34.602	1	< 0.001	0.001

B = B coefficient. SE = standard error. Df = degrees of freedom. OR = Odds Ratios. Dependent variable: HRESW Scale. Sig = Bilateral significance

## Discussion

It has been pointed out that the application of human rights in the processes carried out by mental health professionals makes the recovery process more effective by reducing stigma and improving the attitude towards learning concepts related to mental health (Gill et al., 2024). Despite the above, difficulties persist in engagement with human rights in the training processes of these professionals, even when this rights-based approach underpins their professional training (Mukhalalati & Taylor, 2019). Therefore, the present research shown how engagement with human rights is related to attitudes of authoritarianism, benevolence, social restriction towards people with mental disorders, and with socioeconomic status, in Colombian medical and social work students. This confirms the hypothesis raised by the present study and allows human rights training to focus on elements that go beyond theory.

Smith & Williams (2021) have highlighted motivation aspects related to the application of human rights in healthcare practices. This reaffirms the present study in its relationship between human rights engagement, stigma, and the importance given to mental health. Similar occurred with the background of a previous diagnosis, which also impacted the attitude toward mental disorders and some variables of the notion and commitment to human rights. This agrees with findings by Ofei-Dodoo et al. (2019), who describes that empathy could be higher in health professionals. A better attitude towards mental health care has also been noted in students with some type of disability and with the socioeconomic condition (Mayer et al., 2023), as suggested by the present study. Other studies have indicated that women have a greater commitment to human rights and less stigma towards mental disorders (Lewis, 2023). However, this was not the case in the present research.

Other studies have described that, despite mental health training, mental disorders are often stigmatized more than physical disorders (Riffel & Chen, 2020). Among the mechanisms of stigma, it has been proposed that this negative perception could be due to knowledge problems, attitudinal problems, and behavioral problems or discrimination (Thornicroft et al., 2007). The way mental health personnel relate to people with mental disorders may also depend on intrinsic factors of the professional from previous experiences, their philosophy of care or socialization related to gender, culture and medical training (Decety, 2020). In this sense, it has been described that human rights training has been shown to decrease this stigma (Gill et al., 2024).

In Latin American contexts, better implementation and greater acceptability of strategies that have human rights as a frame of reference have been shown (Agudelo-Hernández et al., 2024; Hernández et al., 2024). In others low- and middle-income countries, the main core component of strategies to reduce stigma towards mental illness was to promote knowledge among health professionals (Clay et al., 2020). However, few strategies indicate human rights engagement as an objective (Clay et al., 2020).

Although education on mental illness has been described as increasing general knowledge, creating more awareness of the problems and needs of people with mental illness, and increasing empathy (Riffel & Chen, 2020), the present study found no differences between levels of training and stigma towards people with mental disorders. In this regard, other studies have raised the need to strengthen human rights training in mental health-related professions (Schonholz et al., 2020), as insufficient education resulted in low confidence in the skills to provide treatment to people with mental illness (Waugh et al., 2017).

The need for a lack of solid designs in educational strategies for mental health professionals to reduce stigma and promote human rights is noted, as well as for increasing studies in low- and middle-income countries. This coincides with systematic reviews that have addressed the core components of strategies to reduce stigma (Clay et al., 2020) and with global guidelines, such as the Lancet Commission on Mental Health and Sustainable Development and the proposal for the future of mental health training (Patel et al., 2018).

### Implications

While knowledge is necessary to impact health systems through education, experience would suggest that they are not enough on their own (Mukhalalati & Taylor, 2019; Smith & Williams, 2021). Overcoming these challenges implies a significant change at the level of the educational structure itself, where changes in attitude are also mobilized, and the beliefs and cultural factors of the students are considered (Cornett et al., 2023; Gill, 2018; Gronholm et al., 2023; Moudatsou et al., 2020).

Promoting global health equity requires the use of tools that aim to eliminate forces that threaten human dignity, with the aim of ensuring health as a human right for all. In care processes, a combination of individual experience, critical reflection, dialogue, holistic orientation, context awareness, and authentic relationships is required. These transformative experiences create conditions for the emergence of critical awareness, involving recognizing people seeking care as subjects of rights (Gill et al., 2024), which also impacts greater acceptability of proposed interventions from the health system (Agudelo-Hernández et al., 2024).

### Limitations

It is important to consider other factors that influence interactions between professionals and people with mental disorders. It is important to investigate unrecognized or unidentified prejudices towards people with mental illness, which may be part of structural stigma and which may prevent human rights from being put into practice. Likewise, deficiencies in knowledge, training, resources available to the health system and behavioral responses of students towards people with mental disorders should be better investigated. It is recognized as a limitation that other professions related to addressing mental health have not been included. Future studies could address experimental designs to determine the impact that human rights training strategies have on these professions.

In conclusion, the present study shows correlations between the perception of people with mental disorders, attitudes toward mental disorders in clinical teaching, and human rights in academic processes, both theoretically and practically. At the explanatory level, it is found that variables such as socioeconomic status, human rights notions, as well as authoritarianism, benevolence, and mental health ideology, could explain the variance of human rights engagement in social work and medicine students. The need to build pedagogical strategies that allow internalizing human rights for mental healthcare and mobilizing the attitudes of future mental health professionals is highlighted.

## Data Availability

No datasets were generated or analysed during the current study.
